# Exploring PROTAC Cooperativity with Coarse-Grained Alchemical Methods

**DOI:** 10.1021/acs.jpcb.2c05795

**Published:** 2023-01-06

**Authors:** Huanghao Mai, Matthew H. Zimmer, Thomas F. Miller

**Affiliations:** Division of Chemistry and Chemical Engineering, California Institute of Technology, Pasadena, California91125, United States

## Abstract

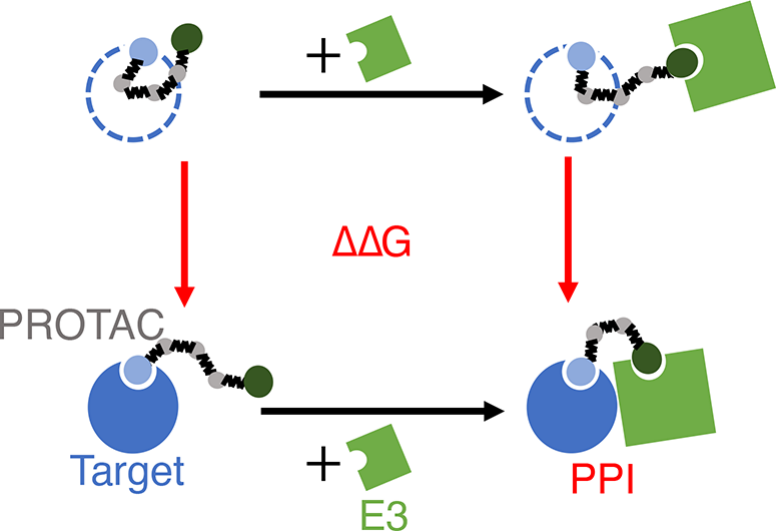

Proteolysis targeting chimera (PROTAC) is a novel drug modality that facilitates the degradation of a target protein by inducing proximity with an E3 ligase. In this work, we present a new computational framework to model the cooperativity between PROTAC–E3 binding and PROTAC–target binding principally through protein–protein interactions (PPIs) induced by the PROTAC. Due to the scarcity and low resolution of experimental measurements, the physical and chemical drivers of these non-native PPIs remain to be elucidated. We develop a coarse-grained (CG) approach to model interactions in the target–PROTAC–E3 complexes, which enables converged thermodynamic estimations using alchemical free energy calculation methods despite an unconventional scale of perturbations. With minimal parametrization, we successfully capture fundamental principles of cooperativity, including the optimality of intermediate PROTAC linker lengths that originates from configurational entropy. We qualitatively characterize the dependency of cooperativity on PROTAC linker lengths and protein charges and shapes. Minimal inclusion of sequence- and conformation-specific features in our current force field, however, limits quantitative modeling to reproduce experimental measurements, but further development of the CG model may allow for efficient computational screening to optimize PROTAC cooperativity.

## Introduction

Proteolysis targeting chimera (PROTAC) has emerged as a promising drug modality that elicits protein degradation by hijacking the ubiquitin–proteasome system (UPS), a major regulatory component of cells. In the UPS pathway, E3 ligases transfer ubiquitins onto aberrant proteins to mark them for degradation by proteasomes. A PROTAC molecule exploits this pathway with two binding moieties that tether the target protein and an E3 ligase together. The tethered target protein thus becomes a neo-substrate of the E3 ligase and is subsequently ubiquitinated for proteasomal degradation. PROTACs require a lower dose than conventional small-molecule inhibitors because of their catalytic nature and they have the potential to target the undruggable proteome.^1,2^ Since the first proof-of-concept in 2001,^3^ the number of proteins successfully degraded by PROTACs has grown rapidly, and examples of such proteins include kinases and gene regulators that are implicated in cancer. As of 2021, at least 13 PROTACs are in or approaching clinical trials.^4^

Despite increasing applications, there is a lack of guidance on designing PROTACs due to the unique mode of action.^5−7^ In particular, a critical step in the degradation process is the formation of the ternary complex of target–PROTAC–E3. The ternary complex involves molecular interactions beyond the binary bindings between the two warheads of a PROTAC and the two proteins. The selectivity^8−10^ and stability^11−14^ of the ternary complex can both be improved through favorable protein–protein interactions (PPIs) between the target protein and the E3 ligase. For certain targets, the degradation outcome can be very different depending on whether cereblon (CRBN) or von Hippel–Lindau (VHL), the two most heavily used E3 ligases, more efficiently and selectively form a productive complex with the target.^11,15−17^ As more warheads for E3 ligases are designed,^18−21^ choosing which of the more than 600 E3 ligases in humans^22^ optimally interact with the target protein will become important.^23,24^ While PPIs depend on the sequences and the structures of the proteins, PROTACs can also modulate the PPIs by restricting the distance and relative orientation between the target and the E3 ligase, effectively changing the entropic component of PPIs.

Because of this three-body interplay and the transient nature of the ternary complex, a complete characterization of the PPIs as a function of the PROTAC, the target protein, and the E3 ligase is intractable. A few proteomics studies^16,17,25^ on kinase degradation have used PROTACs with promiscuous warheads such that the PROTAC-induced PPIs differentially affect the degradation outcome of hundreds of proteins. These studies reported the fold change of protein abundance due to PROTAC treatment, but analysis can be complicated by secondary interactions^24^ and numerous other factors such as the permeability of the PROTAC, half-lives of the target proteins, cellular localization, and reactions downstream of ternary complex formation.^26^ Other studies^8,9,27−30^ have focused on specific target-E3 pairs and examined the effect of changing PROTAC properties such as the linker length. They measured the difference in the strength of PROTACs binding to the target or the E3 ligase due to the presence of the other protein. This difference, termed binding cooperativity, reflects the strength of PROTAC-mediated PPIs. However, few generalizable patterns have emerged and systematic experimental characterizations remain scarce.

Computational modeling based on docking or atomistic molecular dynamics (MD) has complemented experimental work^9,29^ and displayed promising future prospects, but there are several limitations to current methodologies. Although standard docking protocols do not handle three-body problems, several workflows have been adapted ad hoc for PROTAC.^31−35^ Docking studies rank ternary complex conformations by scoring functions biased for naturally evolved PPIs and benchmark against the few crystal structures of PROTAC-induced ternary complexes.^36−38^ The results can be inaccurate as PROTAC-induced PPIs are non-native and exhibit plasticity.^9,39^ In contrast, atomistic MD is physically grounded to capture non-native PPIs. However, the size of the ternary complex modeled at an atomistic resolution significantly limits the time scale of simulations, such that naively simulating PPIs can be prohibitively slow. Sophisticated enhanced sampling techniques and distributed computing are needed to sample an ensemble of low-energy conformations that are consistent with experimental data.^40^ Due to the difficulties in modeling the ternary complex, direct calculation of the binding cooperativities was not attempted until two recent studies^41,42^ that explored the molecular mechanics with the generalized Born and surface area continuum solvation (MM/GBSA).

Here, we seek an orthogonal approach that combines coarse-grained MD (CGMD) and alchemical free energy calculation methods to study PROTAC cooperativities. On the spectrum of computational tools, docking and atomistic MD are positioned at the empirical and first-principle ends, respectively, and finding a compromise in the middle of this spectrum is a promising direction. Compared to atomistic modeling, coarse-graining reduces the effective size of the model and smoothens the energy surface, enabling simulations at a much longer time scale necessary for the PROTAC-mediated complexes. While CGMD may struggle to recapitulate the molecular basis of lock-and-key bindings, such strong and specific interactions are less imperative in non-native PPIs induced by PROTACs. Moreover, PROTAC binding reduces the ways proteins can interact with each other, differentiating and simplifying the problem studied here from the formidable task of modeling general protein–protein binding. In docking, such constraints are challenging to incorporate into the scoring functions and are approximated through separate steps to filter compatible PPI poses and PROTAC geometries. While CGMD excludes many degrees of freedom from the PROTAC, proteins, and solvent entropy, this effect of configurational entropy on PPIs from PROTAC mediation can be directly captured. Finally, we calculate binding energies using alchemical methods, which circumvents the computational challenge of directly sampling binding and unbinding events between the PROTAC and proteins. We demonstrate the computational amenity of an unconventional application of alchemical methods motivated by the PROTAC systems, and take advantage of the physical interpretability of the CGMD + alchemical approach to explore the principles of PROTAC binding cooperativity.

## Methods

### CGMD Setup of PROTAC–Protein Complexes

The binary and ternary PROTAC-protein complexes are coarse-grained at two resolutions to efficiently sample complex conformational changes while retaining sufficient details for structural insight. Specifically, a major focus of this work is to characterize the entropic effect of the length of PROTACs on the strength of induced PPIs, necessitating modeling the PROTAC linker at a higher resolution than the rest of the system. Proteins are coarse-grained by mapping every three amino acids onto a large bead of σ = 0.8 nm diameter, which is approximately the Kuhn length of polypeptides.^43−46^ Binding moieties at the two ends of a PROTAC are each represented by a large bead, whereas the linker region is modeled as a Gaussian chain at the resolution of a PEG unit (σ_*s*_ = 0.35 nm)^47^ or three heavy atoms. Several experimental works that used flexible linear linkers motivate our modeling approach for the PROTAC linker, including Chan et al.^28^ where an alkane linker was varied in step sizes of our linker beads and Zorba et al.^29^ where a PEG linker is modified at smaller length steps such that linker lengths ranging from 1 to 6 σ_*s*_ in our modeling correspond to the PROTAC (1), (3), (5), (6), (8), and (10).

A minimal force field is used to describe the internal and interactive forces, and a full description can be found in the Supporting Information (Section S1). The three-dimensional structure of a protein is maintained by a bottom-up fitted elastic network model (Figure S2), which allows conformational flexibility.^48,49^ Protein beads can have additional properties to describe PPIs beyond volume exclusion (Figure S1). When modeling electrostatic interactions, for example, a protein bead has the net charges of the triplet of residues that it is coarse-grained from. PROTACs are modeled as Gaussian polymers with volume exclusion, and the warhead beads are attached to the binding pockets of proteins through harmonic springs. Modeling PROTAC interactions beyond warhead binding is out of the scope of this work. Thus, under current setup, PROTAC beads have 0 charge and no affinity to any other beads.

**Figure 1:**
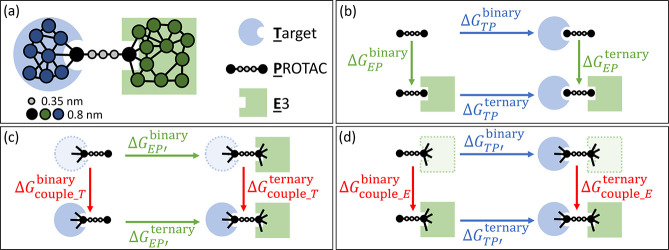
Schematic of the simulation setup for PROTAC-mediated complexes. (a) Target–PROTAC–E3 ternary complex is initialized with a fully extended PROTAC as drawn. The proteins are coarse-grained at the resolution of three amino acids per bead, approximately 0.8 nm. PROTAC warhead beads are represented by beads of the same size, whereas the linker is coarse-grained at a higher resolution.(b) PPIs affect how cooperative target–PROTAC and PROTAC–E3 bindings are and are reflected in the free energy difference between PROTAC–E3 binding with and without the target . This free energy difference, ΔΔ*G*, can also be obtained by comparing target–PROTAC binding with and without the E3  as shown by the thermodynamic cycle. Under the alchemical setup, ΔΔ*G* can be alternatively obtained by the free energy difference between the red vertical processes, which represent coupling the target ( in (c)) or the E3 ( in (d)) to the PROTAC and the PROTAC prebound to the other protein. In the initial states in (c) and (d), the dotted lines represent the target or the E3 whose interactions with the rest of the system are turned off except for the harmonic constraints (black lines) to the PROTAC warhead.

The orientation between the E3 ligase and the target protein is initialized such that the two binding pockets face each other, with a fully extended PROTAC tethering in between (Figure 1a). The binding moiety beads of PROTAC are placed at the center of each binding pocket, which is defined by the residues within 4 or 5 Å from the PROTAC warhead in experimental structures. Thus, setting up the initial coordinates of a ternary complex requires the following inputs: structures of each protein, residues at the two PROTAC binding pockets, and the length of the PROTAC linker. To calculate the difference in PROTAC binding energies due to PPIs, simulations of binary target/E3-PROTAC complexes are also needed. Binary complexes are prepared by removing a protein from the initialized ternary complex.

### Thermodynamic Framework of Alchemical Perturbation

The binding cooperativity of a PROTAC is mathematically defined as exp(ΔΔ*G*/*RT*), where *R* is the gas constant, *T* here refers to the temperature in the context of an energetic scale and refers to the target protein elsewhere, ΔΔ*G* = Δ*G*_*TP*_^binary^–Δ*G*_*TP*_^ternary^, and Δ*G*_*TP*_^ternary^ and Δ*G*_*TP*_^binary^ are the free energies of the PROTAC (*P*) binding to the target protein (*T*) with and without the presence of the E3 ligase (*E*). Because of the thermodynamic cycle (Figure 1b), the same ΔΔ*G* can be obtained from Δ*G*_*EP*_^binary^ – Δ*G*_*EP*_^ternary^. Favorable PPIs stabilize the ternary complex and facilitate PROTAC binding to both proteins. Thus, they lower Δ*G*_*TP*_^ternary^ and Δ*G*_*EP*_^ternary^, which leads to larger ΔΔ*G* and more positive cooperativity.

Alchemical free energy calculation methods exploit alternative thermodynamic cycles to obtain ΔΔ*G* without simulating binding and unbinding processes. For simplicity, in this work, all ΔΔ*G*s are calculated using the cycle in Figure 1c, which we describe in detail here, but one should arrive at the same result using the mirroring cycle in Figure 1d. By the definition of a thermodynamic cycle, we have , where  and  represent the free energies of coupling *T* to *P* and to the target–PROTAC bound complex *EP*. In the initial states of both coupling processes (vertical processes in red in Figure 1c), *T* is bound to *P* but is a dummy molecule at an ideal state. Specifically, multiple harmonic springs connect the binding pocket beads in *T* to the warhead bead of *P*, and *T* itself is an elastic network model consisting of only harmonic springs. All other interactions between *T* and the rest of the system, whether *P* or *EP*, are turned off. Coupling *T* simply means turning on these intermolecular interactions, while the binding pocket springs remain unperturbed.

Attaching a dummy *T* instead of having *T* dissociated results in a systematic error in the horizontal free energies of *EP* binding ( and  in Figure 1c) such that the ΔΔ*G* is unaffected. This is because the attachment of dummy *T* occurs via only one bead on *P*, except which there are no other force field terms involving both physically present beads and dummy beads. In the configurational partition function, energy terms describing the geometries of the physically present part of the system can therefore be separated from the term involving the dummy *T* and the attachment junction. The latter term is the same whether the physically present part is *P* or *EP*, such that the unphysical contribution from attaching dummy *T* cancels out in ΔΔ*G*.

### Free Energy Calculations

**Figure 2:**
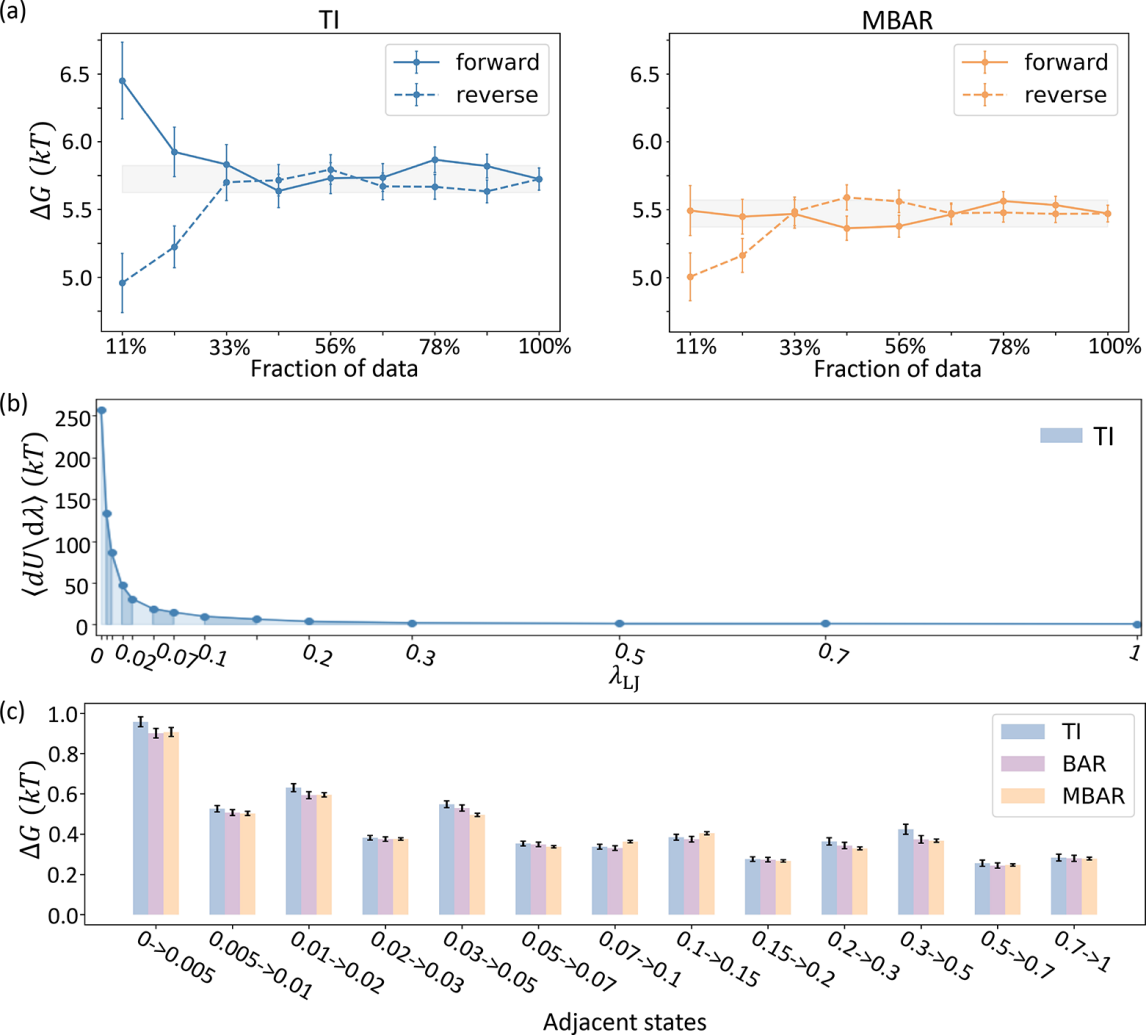
Calculation of Δ*G*^ternary(sterics)^ by alchemical perturbation of BTK in the ternary complex of BTK-PROTAC (10)-CRBN. (a) TI and MBAR both reach apparent convergence in the time-forward and time-reversed directions with no pathological signs. The gray band in each panel represents the final estimation using 100% data ± 0.1 *kT* as a threshold for error tolerance, where *k* is the Boltzmann constant. (b) TI estimation is shown as the blue area under the curve of ⟨*∂U*/*∂λ*⟩. (c) TI, BAR, and MBAR agree for all intermediate Δ*G*s between adjacent states. All error bars of computational results here and in subsequent figures represent ±1 std. Color coding for TI, BAR, and MBAR results are the same in subsequent figures unless otherwise stated.

Alchemically changing a protein from a dummy state to full coupling involves turning on the interaction potentials between the protein and the rest of the system in the force field. The interactions are turned on in stages by sequentially scaling each kind of interaction potential using a coupling parameter λ. Intramolecular potentials (e.g., the elastic network model of each protein) and intermolecular potentials not perturbed at the current stage are unaffected by the λ scaling. For the electrostatic potential, the start state (no electrostatics) and the end state (full electrostatics) correspond to λ_elec_ = 0 and 1 respectively. Intermediate states are interpolated such that the potential is defined as . For numerical stability, the electrostatic potential is only perturbed in the presence of volume exclusion,^50,51^ which is modeled by Weeks–Chandler–Andersen (WCA) potential. To turn on Lennard-Jones (LJ) or variants of LJ potentials (e.g., WCA), a soft-core scaling^52^ with λ_LJ_ is used for numerical stability:
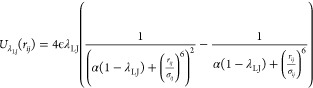
where α = 0.5, *r*_*ij*_ is the distance between beads *i* and *j*, and σ_*ij*_ is the sum of the radii of beads *i* and *j*. The number of intermediate states and the spacing of the coupling parameter values depend on the difficulty to obtain converged free energy calculations. For the electrostatic potential, a linear pathway where λ_elec_ ranges from 0 to 1 with a step size of 0.125 is a simple and effective approach. For LJ and related potentials, because most of the free energy changes occur near the start state of λ_LJ_ = 0 (Figure 2b,c), we introduce intermediate states at λ_LJ_ = 0.005, 0.01, 0.015, 0.02, 0.04, 0.06, 0.08, 0.1, 0.2, 0.3, 0.5, 0.7, and 0.9.

The Δ*G* of turning on each kind of interaction is calculated using thermodynamic integration (TI),^53^ Bennett acceptance ratio (BAR) method,^54^ and the multistate BAR (MBAR) method.^55^ TI and BAR/MBAR are distinct formulations for free energy calculations, and we verify that these methods converge to similar values. The system in CGMD is evolved using overdamped Langevin dynamics with a diffusion coefficient of 253 nm^2^/s and a time step of 30 ns for stable integration. At each state, at least 64 trajectories of 6 s long are generated to sample the conformations of the complexes. After collecting the samples from trajectories, postprocessing involves calculating ∂*U*/∂λ and Δ*U*_*ij*_ for all *i*, *j* = 1, 2, ..., *K* states as inputs for TI, BAR, and MBAR.

## Results and Discussion

### Alchemical Perturbation of Protein Domains Is Feasible with CGMD

The binding cooperativity of PROTAC due to PPIs is a unique challenge that calls for an unconventional application of alchemical free energy calculation methods. Alchemical methods are mainly used to determine the binding energies between small-molecule ligands and proteins, and typically no more than 10 heavy atoms are perturbed for efficient and accurate calculations. In protein–protein binding, recent applications and development focus on quantifying the relative free energy changes from small-scale perturbations such as mutations of single residues.^56−60^ To our knowledge, the only case that alchemically calculates PPIs in a three-body setting compares how analogs of inhibitors change aberrant multimerization of the HIV-1 integrase.^61^ Their proposed thermodynamic framework involves calculating the relative free energy difference by perturbing small molecules that directly participate at a fixed PPI interface. This framework is more readily extendable to molecular glues that modulate PPIs in a similar way. PROTACs, however, due to a more modular design, are typically larger linear molecules. The flexibility of the linker is often nontrivial, such that the two proteins cannot be kept bound at a fixed interface. This configurational entropic concern necessitates an unusually large perturbation at the scale of a protein rather than a small molecule to calculate the binding cooperativity, testing the computational limit of alchemical methods.

To explore the feasibility of the CG alchemical approach, we calculate the free energy of turning on the steric repulsions between a target protein and a PROTAC–E3 complex (Δ*G*^ternary(sterics)^) in the absence of other intermolecular potentials. We choose Bruton’s tyrosine kinase (BTK) as the target (only the kinase domain modeled), CRBN as the E3, and the PROTAC (10) from ref (29), which are respectively modeled by 87, 124, and 8 beads in the CG model. Together they form the largest target–PROTAC–E3 complex simulated in this work. We compare the calculations using different percentages of the simulation data collected in the time-forward and time-reversed directions. The calculated values of Δ*G*^ternary(sterics)^ plateau starting around the midpoint of the simulation time, indicating numerical convergence (Figure 2a). The time-forward and -reversed estimations are within 1 standard deviation (std) at the midpoint, and the time-reversed estimations remain stable after the midpoint. The observed behavior of the estimates over time suggests that unequilibrated samples at the beginning of the trajectories have been removed, and the remaining frames sample from similar distributions rather than distinct metastable states with slow transition rates.^51^

Three methods, TI, BAR, and MBAR are used to separately estimate the free energies. The accuracy of all three methods depends on the number and the spacing of alchemical states. BAR and MBAR reweight conformations sampled from one state by their probability in another state to estimate the free energy differences. Having similar probability distributions between states (i.e., phase space overlap) is therefore critical to the estimation. Unlike BAR/MBAR, TI estimates the free energies by numerically integrating ⟨∂*U*/∂λ⟩, the ensemble average of the derivative of the potential energy *U* along the alchemical pathway defined by λ. Depending on the curvature of ⟨∂*U*/∂λ⟩, choices of intermediate states specified by λ and the integration scheme together introduce integration errors in addition to the statistical errors in estimating the ensemble average per state. We choose an alchemical pathway that involves 12 intermediate states in addition to the start and end states, such that , where  is the free energy of changing the WCA potential between neighboring states λ_*i*_ and λ_*i*+1_. With a total of 14 states unevenly spaced, the phase space overlap between neighboring states is sufficient (Figure S3) for efficient reweighting-based estimations. For TI, the trapezoid rule of numerical integration is used for its simplicity and robustness. Although the quadrature errors result in a slight overestimation of Δ*G*^ternary(sterics)^, the *∂U*/*∂λ* curve is sufficiently smooth such that TI and MBAR largely agree. In addition to the global agreement on Δ*G*^ternary(sterics)^, TI, BAR, and MBAR also locally agree with each other on all  along the alchemical pathway (Figure 2c). We emphasize that TI and BAR/MBAR rely on distinct types of input data and processing procedures, and their consistency even at the most granular level of calculations further validate our CG alchemical approach.

Analyses of estimations over simulation time and using different free energy calculation methods indicate that convergence of perturbing a protein can be achieved within reasonable computation time, significantly pushing the boundaries of applying alchemical methods. As parallelization can be done over the alchemical states and over trajectories for each state, the time to run one trajectory is the main limiting factor in the wall-clock computation time of applying our method. Criteria to determine how long a trajectory should be run are described in the Supporting Information (Section S2). For this work, depending on the size of the system, 3–14 CPU hours per trajectory of ternary complexes are sufficient.

### Minimal Force Field Captures Entropic Effects in PROTAC-Mediated PPIs

**Figure 3:**
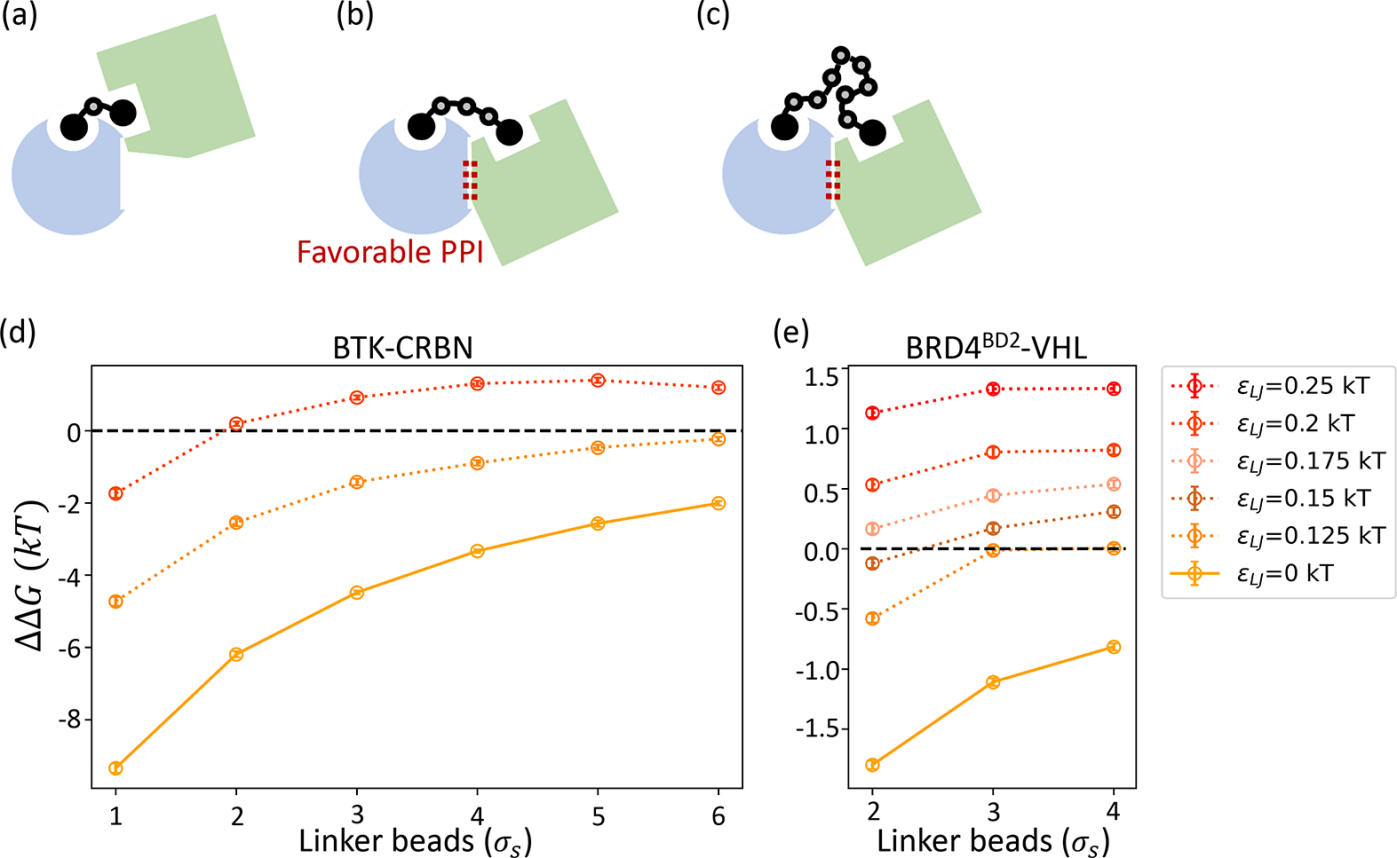
PROTAC linker length changes ΔΔ*G* through modulating the effective strength of PPIs. The top three schematics illustrate the scenarios where a PROTAC linker is (a) too short to enable favorable contacts between the target (blue) and the E3 (green), (b) at an optimal length, and (c) sufficiently long but less frequently in a configuration that induces weak favorable PPIs (red dots). The ΔΔ*G* trends over PROTAC linker lengths are calculated for two target-E3 pairs, (d) BTK-CRBN and (e) BRD4^BD2^-VHL, under varying strengths of nonspecific attractions between proteins. The solid lines represent the baseline ΔΔ*G* trends where only volume exclusion is modeled between the two proteins, and the dotted lines show the trends where nonspecific attractions are added. The strengths (ϵ_LJ_) of attractions are indicated by different colors. Higher ϵ_LJ_ represents stronger attractions, and the baselines can also be considered as results at ϵ_LJ_ = 0. Results at ϵ_LJ_ = 0.125 and 0.2 *kT* are plotted for BTK-CRBN and results at ϵ_LJ_ = 0.125, 0.15, 0.175, 0.2, and 0.25 *kT* are plotted for BRD4^BD2^-VHL. All calculations shown are obtained using MBAR, and results using TI and BAR are superimposed in Figure S5.

Encouraged by the proof-of-concept calculations above for Δ*G*^ternary^, we also calculate Δ*G*^binary^ and complete our calculations for the ΔΔ*G* of the thermodynamic cycle. We follow the sign convention of ΔΔ*G* such that a positive value represents positive cooperativity. The BTK-CRBN system modeled here has been experimentally shown to lack large cooperativity, and introducing PROTACs in hydrogen/deuterium exchange experiments did not reveal significant profile changes that would indicate the presence of stable PPIs. As the starting point for our method development, we focus on this system due to its apparent simplicity and the availability of experimental characterization over a large range of PROTAC linker lengths. We characterize ΔΔ*G* changes over PROTAC lengths because this relies on capturing the fundamental physics of the tertiary interactions (Figure 3a–c) rather than sequence- or conformation-specific properties.

Two force field setups are used to describe PPIs and the resulted ΔΔ*G* trends over PROTAC linker lengths are compared. In the first setup, we calculate the baseline ΔΔ*G* in the absence of PPIs other than volume exclusion. In the second setup, nonspecific attractions between BTK and CRBN beads are added and explored at two strengths. The intrinsic PPIs without PROTAC mediation should be weak such that in the limit of infinite linker length the ΔΔ*G* is negligible. The attenuation of weak PPIs with increasing PROTAC linker lengths originates from configurational entropy. As the PROTAC becomes longer, it experiences a greater loss of configurational freedom upon binding to proteins to induce PPIs (Figure 3b and c), incurring an entropic cost. We examine this configurational entropic effect by modeling ΔΔ*G* at linkers ranging from 1 to 6 beads (σ_*s*_) long, which correspond to approximately 3.5 Å to 21 Å.

In the first setup, the steric cores of the proteins should penalize PROTAC binding and result in negative cooperativities. This is because some conformations that are accessible to the PROTAC in a binary PROTAC–protein complex become inaccessible in the ternary complex due to steric clashes (Figure 3a). As the linker length increases and steric clashes are attenuated, the cooperativity should become less negative. We verify that such a monotonically increasing trend of negative ΔΔ*G* is obtained in our model (Figure 3d). Steric penalties on ΔΔ*G* are most obvious at the region of short linker lengths (1–3 beads), after which the benefit from extending the linker length becomes increasingly marginal, and we expect that beyond the simulated window of linker lengths, ΔΔ*G* will eventually plateau near 0. This ΔΔ*G* trend is consistent with a recent effort to tabulate PROTAC linker length structure–activity relationships (SAR), which suggests that steric clashes at short linker lengths often result in a steep decrease in activity.^38^

After validating the baseline trend, we next examine how the cooperativity trend is changed by the addition of favorable PPIs through LJ potentials. Increasing the well depth of LJ (ϵ_LJ_) increases the strength of this nonspecific attraction, which is kept weak (Figure S1) to approximate van der Waals forces. At the attraction strength of ϵ_LJ_ = 0.125 *kT*, the ΔΔ*G* curve is elevated compared to the previous curve without attraction (Figure 3d), as favorable PPIs are expected to enhance cooperativity. Nevertheless, at this attraction strength, steric penalties still dominate, and ΔΔ*G*s remains negative. Even though adding an LJ potential brings an additional penalty when beads overlap, shorter PROTACs still benefit more from the attractive part of LJ than longer PROTACs, resulting in a flatter ΔΔ*G* trend as compared with the purely repulsive interactions.

An appropriate combination of repulsive and attractive forces may generate a nonmonotonic ΔΔ*G* trend, such that intermediate linker lengths promote optimal cooperativity by minimizing steric clashes while maximally sampling attractive PPIs.^38^ As the attraction strength increases to ϵ_LJ_ = 0.2 *kT*, intermediate-length PROTACs exhibit not only positive ΔΔ*G*s but the values can be comparable and even slightly higher than that of the longest PROTAC (Figure 3d). Within the limited window of linker lengths, only the initial part of the decaying tail of a nonmonotonic ΔΔ*G* trend is observed. We expect that beyond the simulated window of linker lengths, configurational entropic penalties will continue driving ΔΔ*G* down toward 0.

Experimentally, the linker length at 3 beads uniquely enables weak positive cooperativity for BTK-CRBN, whereas our results at ϵ_LJ_ = 0.2 *kT* remain biased toward favoring longer linkers and are not as sensitive to linker length changes. To see whether these characteristics are specific to the choice of the system, we then examine the ΔΔ*G* trends for a different system (Figure 3e), BRD4^BD2^-VHL, where experimentally, the linker length at 3 beads can also optimize the cooperativity.^28^ Due to the smaller size of the system, we can afford to calculate ΔΔ*G*s at three more attraction strengths. Similar to BTK-CRBN, in the absence of attractions, negative ΔΔ*G* monotonically increases over the linker length, and adding nonspecific attractions results in flatter and higher ΔΔ*G* curves. Within the narrow window of short linker lengths, scanning the attractive strength ϵ_LJ_ from 0.125 to 0.25 *kT*, however, does not recapitulate the optimal linker length at 3 beads. This result suggests that enhancing nonspecific attractions in the minimal model is insufficient to compensate for the steric penalties while remaining sensitive to entropic penalties from the linker length.

We demonstrate that the minimal CG model directly captures configurational entropic effects on weak nonspecific PPIs through analyzing ΔΔ*G* trends over PROTAC linker lengths. Beyond this entropic effect, combining repulsive and attractive interactions at various strengths changes the behaviors of cooperativity trends and can shift the optimal linker length, as shown in BTK-CRBN. Nevertheless, chemically specific interactions or specific sampling of certain PPIs is needed to model optimal positive cooperativity at an experimentally relevant range and resolution of PROTAC linker lengths.

### Electrostatics in PROTAC-Mediated PPIs Exhibit Plasticity

As a step toward more realistic modeling of cooperativity, we seek chemically specific PPIs to include and further explore the BRD4^BD2^-VHL system due to the availability of experimental structural information. Crystal structures of the ternary complexes have revealed specific interactions that are proposed as the molecular basis for the observed positive cooperativity and selectivity against other structural homologues.^8,62^ As shown in the previous subsection, these interactions between proteins cannot be approximated by nonspecific attractions that contribute to the cooperativity with low sensitivity to linker length and no protein sequence dependence.

**Figure 4:**
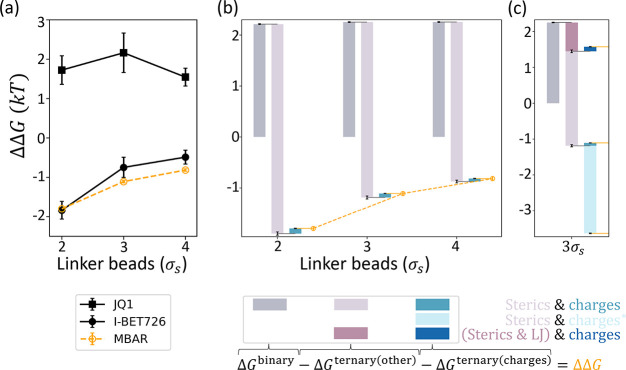
Electrostatic contributions to the cooperativity in the BRD4^BD2^-VHL system are small and context-dependent. All calculations shown are obtained using MBAR, and results using TI and BAR are shown in Figure S6. (a) Calculations of ΔΔ*G*s over PROTAC linker lengths are shown with the experimental measurements^28^ (black) converted to our units. Experimental results at 2, 3, and 4 linker beads correspond to MZ4, MZ1, and MZ2 for PROTACs using JQ1 warhead and MZP-61, MZP-54, and MZP-55 for PROTACs using I-BET726 warhead. (b) Waterfall plot breakdown of ΔΔ*G* calculations. At each linker length, bars in each triplet correspond to Δ*G*^binary^ (gray), −Δ*G*^ternary(other)^ (light purple), and −Δ*G*^ternary(charges)^ (turquoise), and are arranged in a cumulative manner such that the end position marks the resulted ΔΔ*G* (orange). Δ*G*^ternary(other)^ denotes the free energy change of turning on interaction energy components other than the electrostatics, which only include steric repulsions in this panel. (c) ΔΔ*G* breakdowns at linker length 3 under different force field parametrizations are superimposed for comparison. Reducing the screening effect by 10-fold (charges *) significantly increases Δ*G*^ternary(charges)^ (cyan), which leads to a very negative ΔΔ*G*. Introducing nonspecific attractions (ϵ_LJ_ = 0.2 *kT*) not only reduces Δ*G*^ternary(other)^ (dark purple) but also doubles Δ*G*^ternary(charges)^ (steel blue), resulting in a positive ΔΔ*G*.

The structural findings such as salt bridges at the PPI interface and the mutational studies involving charged residues on BRD4^BD2^ and homologues^8^ motivate us to approach chemical specificity through modeling electrostatic interactions. As CGMD uses an implicit solvent, we choose the Debye–Hückel (DH) potential to describe electrostatics in consideration of screening effects under physiological conditions. Within the BRD4^BD2^-VHL system, incorporating charges of protein beads results in a monotonic trend of negative ΔΔ*G*s with increasing linker length, (Figure 4a) similar to the baseline obtained using steric repulsions only (Figure 3e). Since charges are perturbed separately in ΔΔ*G* calculations for numeric stability, in the following discussions, we further investigate our ΔΔ*G* results by isolating the final stage (Δ*G*^ternary(charges)^) in which charges are turned on in the presence of sterics.

Breaking down the ΔΔ*G*s by each energy component shows that at all three linker lengths, Δ*G*^ternary(charges)^ is slightly negative, indicating a mildly favorable process, but the penalty from steric repulsions overwhelmingly dominates electrostatic contributions by an order of magnitude (Figure 4c). As PROTAC linker length increases from 2 to 4 beads, the contribution from Δ*G*^ternary(charges)^ monotonically diminishes. We consider the possibility that the screening of charges is too strong to model more favorable PPIs and tune the screening parameter in the DH potential at the linker length of 3 beads. However, because both the target protein and the E3 ligase have net positive charges, significantly weakening the screening strength leads to a much more unfavorable Δ*G*^ternary(charges)^ (Figure 4c). It is also possible that our level of coarse-graining loses the spatial resolution required for this system to capture detailed interactions like salt bridge formation as observed in the crystal structures.^8,62^

In addition to the small contribution to ΔΔ*G*, Δ*G*^ternary(charges)^ itself exhibits plasticity because conformational sampling at the stage of charge perturbation in alchemical free energy calculations is biased by the potentials turned on in previous stages. The presence of steric repulsions combined with nonspecific attractions at the strength of ϵ_LJ_ = 0.2 *kT*, for example, has doubled the Δ*G*^ternary(charges)^ obtained at the linker length of 3 beads without nonspecific attractions (Figure 4c). Interestingly, this change in Δ*G*^ternary(charges)^ is on top of the favorable contribution from nonspecific attractions in the previous calculation stage (Δ*G*^ternary(other)^) before the inclusion of protein charges. For this particular ternary complex, nonspecific attractions and electrostatic interactions work synergistically.

Our dissection of the electrostatic component in ΔΔ*G* under different simulation setups suggests that a more holistic parametrization is needed to accurately evaluate chemically specific PPIs. For BRD4^BD2^-VHL, incorporating hydrophobic interactions will be of particular interest as there is stacking of hydrophobic residues at the PPI interface observed in the crystal structures.^8,62^ Hydrophobic interactions may also introduce nonadditive free-energy contributions with electrostatics in a similar manner seen with the nonspecific attractions. It is also worth noting that the favorable PPIs revealed by crystal structures are enabled by PROTACs using a JQ1 warhead, which imposes a different linker attachment angle (i.e., exit vector) from an I-BET726 warhead (Figure S7).^28^ Our current force field does not model the PROTAC linker with angular terms to specify the exit vectors, which leads to a ΔΔ*G* trend that matches well with the worse-performing I-BET726 set of PROTACs (Figure 4a). As rigidifying PROTACs is a common strategy to optimize the cooperativity by entropically enhancing certain PPIs,^30,62^ parametrizing linker conformations will improve modeling the specificity in PROTAC-mediated PPIs.

## Conclusions

We explore a novel computational approach to model the binding cooperativity of PROTACs by combining CGMD and alchemical free energy calculations. The plasticity of PROTAC-mediated PPIs motivates an unconventional application of alchemical methods at a perturbation scale that is rarely attempted. We show that with coarse-graining, converged estimates from various free energy calculation methods are attainable within a reasonable amount of computation time. Our results expand the possibility of more creative use of alchemical methods. The feasibility and efficiency of the CG alchemical approach enable us to probe multiple energy components under the alchemical framework and characterize how PROTAC linker lengths modulate PPIs under different setups to produce distinct cooperativity trends. In addition to validating the benefit of using long linkers to avoid steric clashes, we demonstrate with a simple addition of nonspecific attractions between BTK and CRBN that the binding cooperativity can be promoted by shortening the PROTAC linker. Our minimal model is capable of unveiling such changes in cooperativity that are rooted in the configurational freedom of the ternary complexes rather than chemical properties.

Quantitative modeling of the cooperativity, however, remains difficult due to the lack of specificity in the minimal model. Previous studies have recognized the challenges brought by non-native PROTAC-mediated PPIs that are often weak, transient, and pliable, and have called for a paradigm shift toward an ensemble-based characterization beyond a handful of docked or crystal poses.^9,34,39^ While thermodynamic properties such as the binding cooperativity are inherently ensemble-based, we note that both accurate sampling of PPI conformations according to chemical properties and efficient computation to sample a diverse set of conformations are important for calculations. Currently, tuning the strength of nonspecific attractions cannot approximate favorable PPIs while retaining sensitivity to entropic constraints from the PROTAC linker length. Simply adding electrostatic interactions based on amino acid charges proved insufficient to capture the cooperativity trend enabled by JQ1-based PROTACs in BRD4^BD2^-VHL. Additional parametrizations are needed to capture chemically specific PPIs.

Two main avenues are worth exploring for future improvement of our method: PROTAC linker conformations and protein sequence-dependence. Among a myriad of PROTAC properties^6^ that we leave out, structural features such as the exit vector^28^ and the linker rigidity^30,62^ in addition to the linker length can both entropically constrain the sampling of PPIs. Meanwhile, energy components of PPIs other than electrostatic interactions, notably the hydrophobic effects, are currently overlooked. Different energy components may have nonadditive effects in optimizing the absolute cooperativity and relative cooperativities between target homologues such as BRD4^BD2^ and BRD4^BD1^. Although coarse-graining enables efficient computation, parametrization for both directions of force field development will be a major hurdle to overcome. This can be bottom-up using shorter-time scale higher-resolution simulations, similar to that of the CG ENM (Figure S2) in this work. A top-down fitting might also become possible with rapidly growing experimental studies that develop platforms^63^ for empirical SAR of PROTAC linkerology^64,65^ or leverage promiscuous PROTACs and target homologues and mutants to investigate the molecular basis of specificity.^66^

## Supporting Information Available

Description of the force field terms in CGMD, parametrization of CG ENM, analysis of phase space overlap in alchemical free energy calculations of the BTK-PROTAC (10)-CRBN complex, description of postprocessing equilibrated and statistically decorrelated samples from CGMD trajectories for free energy calculations, convergence among TI, BAR, and MBAR for the results shown in Figures 3 and 4, and crystal structures showing the exit vector difference between JQ1 and I-BET726 (PDF)
