# Supplementary material for: Exploring PROTAC Cooperativity with Coarse-Grained Alchemical Methods
Source: J Phys Chem B. 2023 Jan 6;127(2):446–55. doi: 10.1021/acs.jpcb.2c05795 (PMC9869335; doi:10.1021/acs.jpcb.2c05795)
Supplement: Supplementary file 1 — jp2c05795_si_001.pdf [file jp2c05795_si_001.pdf]

Supporting Information: Exploring PROTAC cooperativity with coarse-grained alchemical methods

Huanghao Mai,* Matthew H. Zimmer, and Thomas F. Miller III

Division of Chemistry and Chemical Engineering, California Institute of Technology

E-mail: hmmai@caltech.edu

1 CGMD Forcefield

The complete potential energy function for a ternary complex is

$$\begin{aligned} U\left(\boldsymbol{x};\boldsymbol{b},\boldsymbol{q}\right) = {} & U_{\mathrm{ENM}}\left(\boldsymbol{x}_E\right) + U_{\mathrm{ENM}}\left(\boldsymbol{x}_T\right) + U_{\mathrm{spring}}\left(\boldsymbol{x}_P\right) + U_{\mathrm{WCA}}\left(\boldsymbol{x}_P\right) \\ & + U_{\mathrm{bind}}\left(\boldsymbol{x}_P,\boldsymbol{x}_T;\boldsymbol{b}\right) + U_{\mathrm{bind}}\left(\boldsymbol{x}_P,\boldsymbol{x}_E;\boldsymbol{b}\right) + U_{\mathrm{WCA}}\left(\boldsymbol{x}_P,\boldsymbol{x}_T\right) + U_{\mathrm{WCA}}\left(\boldsymbol{x}_P,\boldsymbol{x}_E\right) \\ & + U_{\mathrm{WCA}}\left(\boldsymbol{x}_E,\boldsymbol{x}_T\right) + U_{\mathrm{elec}}\left(\boldsymbol{x}_E,\boldsymbol{x}_T;\boldsymbol{q}\right) + U_{\mathrm{LJ}}\left(\boldsymbol{x}_E,\boldsymbol{x}_T;\epsilon_{\mathrm{LJ}}\right) \end{aligned} \tag{1}$$

where $\boldsymbol{x}_E$, $\boldsymbol{x}_T$, and $\boldsymbol{x}_P$ indicate the coordinates of the E3 ligase, the target protein, and the PROTAC respectively, $\boldsymbol{q}$ represent the charges of protein beads, and $\boldsymbol{b}$ are indicators of whether protein beads are at the binding pocket or not. All PROTAC beads are modeled with 0 charge and no attraction to the proteins. All parameters and variables are defined using a length scale of the large bead ($\sigma = 0.8$ nm) and an energy scale of $\epsilon = kT$ where k is the Boltzmann constant and $T = 310$ K.

1.1 Internal energy terms

Interactions within a protein are modeled by an elastic network model (ENM) such that every pair of beads within distance R_c is connected by a harmonic spring:

$$U_{\mathrm{ENM}}\left(\boldsymbol{x}\right) = \sum_{(i,j)\in D} k_{\mathrm{spring}}\left(\Delta x_{ij} - d_{ij}\right)^2 \tag{2}$$

where k_{spring} is the spring constant, d_{ij} is the optimal distance between x_i and x_j, and $D = \{(i,j)\,|d_{ij} < R_c\}$. The optimal distance between a pair of beads is its initial distance in the experimental structure. Experimental structures used in this work include VHL (PDB: 5T35[1] chain D), BRD4$^{\mathrm{BD2}}$ (PDB: 5T35[1] chain A), CRBN (PDB: 6BOY[2] chain B), and BTK (PDB: 6W7O[3] chain A), and Schrödinger Maestro[4] is used to fill in missing atoms and perform energy minimization before building the CG ENM. Additional details on the parameterization are described in a separate section below.

PROTAC is modeled as a linear molecule, where adjacent beads are connected by springs ($U_{\text{spring}}(\boldsymbol{x}_P)$) and non-adjacent beads are subjected to steric repulsions ($U_{\text{WCA}}(\boldsymbol{x}_P)$).

1.2 Interaction energy terms

PROTAC-protein interactions consist of binding interactions modeled by springs between a binding moiety bead in the PROTAC and all beads in the corresponding binding pocket ($U_{\text{bind}}(\boldsymbol{x}_P, \boldsymbol{x}_T; \boldsymbol{b})$ and $U_{\text{bind}}(\boldsymbol{x}_P, \boldsymbol{x}_E; \boldsymbol{b})$ in eq.(1)) and steric repulsions ($U_{\text{WCA}}(\boldsymbol{x}_P, \boldsymbol{x}_T)$ and $U_{\text{WCA}}(\boldsymbol{x}_P, \boldsymbol{x}_E)$) between the remaining parts of PROTAC and protein. Steric repulsions in intra-PROTAC, PROTAC-protein, and inter-protein interactions are all modeled by the Weeks-Chandler-Andersen (WCA) potential, a shifted and truncated version of Lennard-Jones (LJ) potential.

Protein-protein interactions are captured by the steric repulsions ($U_{\text{WCA}}(\boldsymbol{x}_E, \boldsymbol{x}_T)$), and depending on the modeling purpose, electrostatics ($U_{\text{elec}}(\boldsymbol{x}_E, \boldsymbol{x}_T; \boldsymbol{q})$) or nonspecific attractions ($U_{\text{LJ}}(\boldsymbol{x}_E, \boldsymbol{x}_T; \epsilon_{\text{LJ}})$). The electrostatic interaction is modeled by a Debye-Hückel (DH) potential. The functional forms and parameterization of both potentials can be found in.[5] When reducing the screening of electrostatics between BRD4^{BD2} and VHL, the Debye length κ is multiplied by 10. The solvent in our system is treated implicitly. Nonspecific attractions aimed at broadly including Van der Waals forces and hydrophobic interactions are modeled by LJ potentials. The strength of the attraction is kept under that of electrostatic interactions (Fig. S1). The well depth of LJ, ϵ_{LJ}, is currently set to be the same for all pairs of beads for nonspecific attraction. For future efforts, minor modifications to the formula[6] and parameterization of ϵ_{LJ} to depend on the Wimley-White hydrophobicity scale, for example, can capture more sequence-specific interactions such as the hydrophobic effects.

1.3 Parameterization of ENM

ENM is a model that represents the tertiary structure of a protein by connecting every pair of protein beads within a certain distance cutoff R_c by a Hookean spring of spring constant

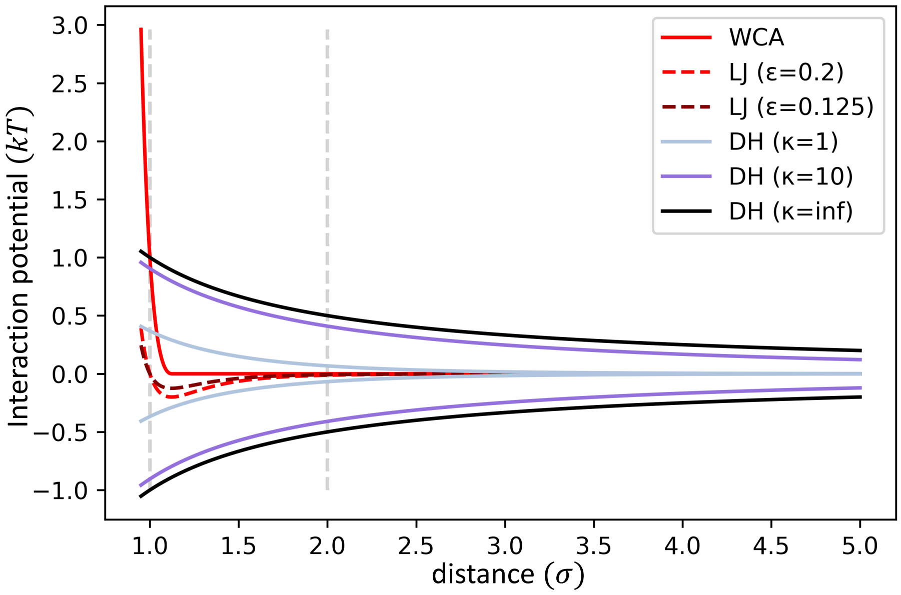

Figure S1: The strengths of various interaction potentials are plotted over the distance between protein beads. The two vertical dashed grey lines bound the distance between 1 and 2 σ. The electrostatic potentials (DH) are plotted for beads with +1 and +1 charges or +1 and -1 charges.

k_{spring}. Despite the simplicity of its parameterization, slow modes in ENM can capture biologically significant conformational changes.[7,8] This structure-based model can also be used in combination with other physics-driven forcefields to model macromolecular complexes. Protein-protein associations and viral capsid assembly have both been successfully modeled by using Elnedyn, an ENM at the resolution of 1 residue per bead,[9] on top of the MARTINI CG forcefield. By fitting to atomistic simulations, Elnedyn preserves both structural properties and dynamics within each protein subunit for the CG simulations.

We follow a similar protocol and fit our CG ENM parameters in eq.(2) to Elnedyn simulations results. Three proteins – IKZF1$^{\text{ZF2}}$ (PDB: 6H0F[10] chain C), BRD4$^{\text{BD1}}$ (PDB: 6BOY[2] chain C), and CRBN (PDB: 6BOY[2] chain B) – are chosen for the fitting to represent the range of protein sizes based on the publicly available crystal structures of PROTAC-mediated ternary complexes. Elnedyn is supported as an option in the MARTINI 2 CG forcefield,[9] and we use the default parameters to generate Elnedyn simulations of these proteins with GROMACS version 5.0.7. Two equilibration stages were run, first at 1 fs

timestep for 50 ps, and then at 10 fs timestep for 1 ns. Then, only the dynamics stage was used for fitting, which was run at 10 fs timestep for 40 ns. Four metrics are used to examine how well a particular combination of k_{spring} and R_c captures information in Elnedyn simulations: the difference of time-averaged root-mean-squared-deviation (ΔRMSD), bead-averaged root-mean-squared-fluctuation (ΔRMSF), Kullback–Leibler (KL) divergence of the RMSD distributions, and the root-mean-squared inner product of the principal components (RMSIP) of the trajectories.

Within a single metric, we usually observe a degeneracy within a certain region of k_{spring} and R_c values (Fig. S2), and this was also observed in Elnedyn fitting to atomistic simulations.[9] This is because increasing either k_{spring} or R_c can increase the stiffness of a protein and, therefore, can compensate for each other to some extent. Nevertheless, despite the degeneracy, given the wide range of protein sizes, there is no single combination of k_{spring} and R_c values that works best for all three proteins. We chose $k_{\text{spring}} = 100\epsilon/\sigma^2$ and $R_c = 2.0\sigma$ as they are near the optimal degeneracy region under most metrics and consistent with the values of Elnedyn parameters ($k_{\text{spring}} = 124.25\epsilon/\sigma^2$ and $R_c = 1.125\sigma$). This combination of k_{spring} and R_c was selected without a global optimization function that combines all four metrics, and should be subjected to finer tuning if a specific system is of interest.

2 Analysis of alchemical free energy calculations

We perform various checks to address two common concerns in alchemical simulations: 1) are there sufficient intermediate states along the alchemical reaction pathway, and 2) are there sufficient samples from each state for accurate free energy calculations. The BTK-PROTAC (10)-CRBN complex is used as an example for the analysis below.

We first validate that there are sufficient intermediate states for a converged estimation of $\Delta G^{\text{ternary(WCA)}}$. The convergence of free energy calculations depends on the overlap of the phase space, i.e. the distribution of sampled conformations, between neighboring states.

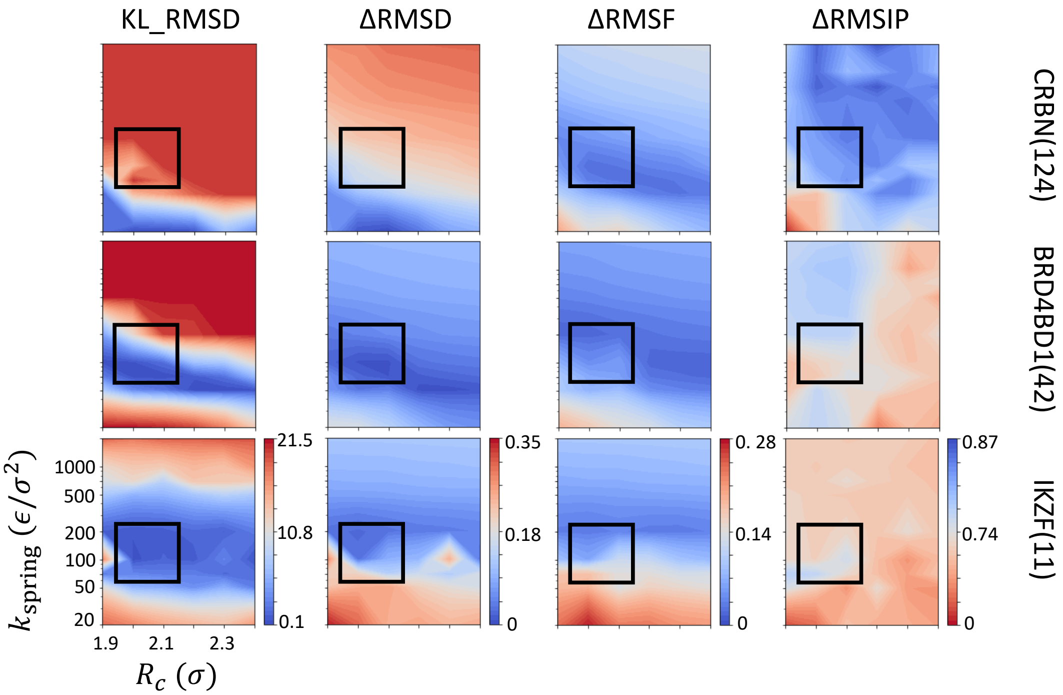

Figure S2: Fitting results of ENM parameters arranged by proteins (rows) and evaluation metrics (columns). Numbers in parenthesis next to protein names are the number of CG beads. For each plot, blue regions indicate k_{spring} and R_c values that result in good fitting, and red regions indicate significant differences between our simulations and Elnedyn simulations. Each column shares the same colorbar range. In general, the boxed regions around $k_{\text{spring}} = 100\epsilon/\sigma^2$ and $R_c = 2.0\sigma$ has good fitting.

Substantial overlap is achieved when the neighboring states are similar, which requires a fine spacing of the coupling parameter values. In practice, distributions of quantities such as ΔU and $\partial U/\partial\lambda$ that are directly involved in free energy estimations are often treated as proxies for the high-dimensional phase space.[11] The similarity between distributions is quantified by KL divergence, where 0 indicates identical distributions and $\gg 1$ suggests concerning differences. Based on this metric, all neighboring states have substantial overlap, as the Kullback–Leibler (KL) divergence values of ΔU and of $\partial U/\partial\lambda$ distributions both stay below 1 (Fig. S3a).

Bennett's overlapping histogram[12] provides another qualitative test for the overlap of ΔU distributions. The difference between $g_{\lambda_{i+1}}(\Delta U_{\lambda_i,\lambda_{i+1}}) = P_{\lambda_i}(\Delta U_{\lambda_i,\lambda_{i+1}}) + (1-C)\,\Delta U_{\lambda_i,\lambda_{i+1}}$ and $g_{\lambda_i}(\Delta U_{\lambda_i,\lambda_{i+1}}) = P_{\lambda_{i+1}}(\Delta U_{\lambda_i,\lambda_{i+1}}) - C\Delta U_{\lambda_i,\lambda_{i+1}}$ is plotted over $\Delta U_{\lambda_i,\lambda_{i+1}}$ values, where C is an arbitrary constant between 0 and 1 and $P_{\lambda_i}(\Delta U_{\lambda_i,\lambda_{i+1}})$ and $P_{\lambda_i}(\Delta U_{\lambda_i,\lambda_{i+1}})$ are the distributions of $\Delta U_{\lambda_i,\lambda_{i+1}}$ obtained by sampling from neighboring alchemical states λ_i and λ_{i+1} respectively. Continuous oscillations of $g_{\lambda_{i+1}}(\Delta U_{\lambda_i,\lambda_{i+1}}) - g_{\lambda_i}(\Delta U_{\lambda_i,\lambda_{i+1}})$ around the estimated $\Delta G_{\lambda_i,\lambda_{i+1}}$ over a range of $\Delta U_{\lambda_i,\lambda_{i+1}}$ values suggests good overlap (Fig. S3b).[13] For states of higher λ_{LJ} values, higher energetic penalty of steric repulsions prevents sampling over a wide range of ΔU values, but the KL divergence and visualization of the distributions (Fig. S3a,c) both indicate the quality of the overlap.

Next, we examine sampling within each state. For each state, a simulation needs to be post-processed to discard the initial unequilibrated part and then subsampled to obtain de-correlated data for accurate uncertainty quantification of the free energy estimation. Thus, the length of the simulations is dictated by the equilibration time, autocorrelation time, and the number of de-correlated samples needed for converged estimations. We examine the values of ΔU, $\partial U/\partial\lambda$, and other collective variables over the simulation time, which typically equilibrate after 0.9 s (Fig. S4a). To find out the decorrelation time, we discard the initial 0.9 s of simulations and plot the autocorrelation functions of these variables over different time lags up to half of the simulation time to ensure that the autocorrelation is calculated

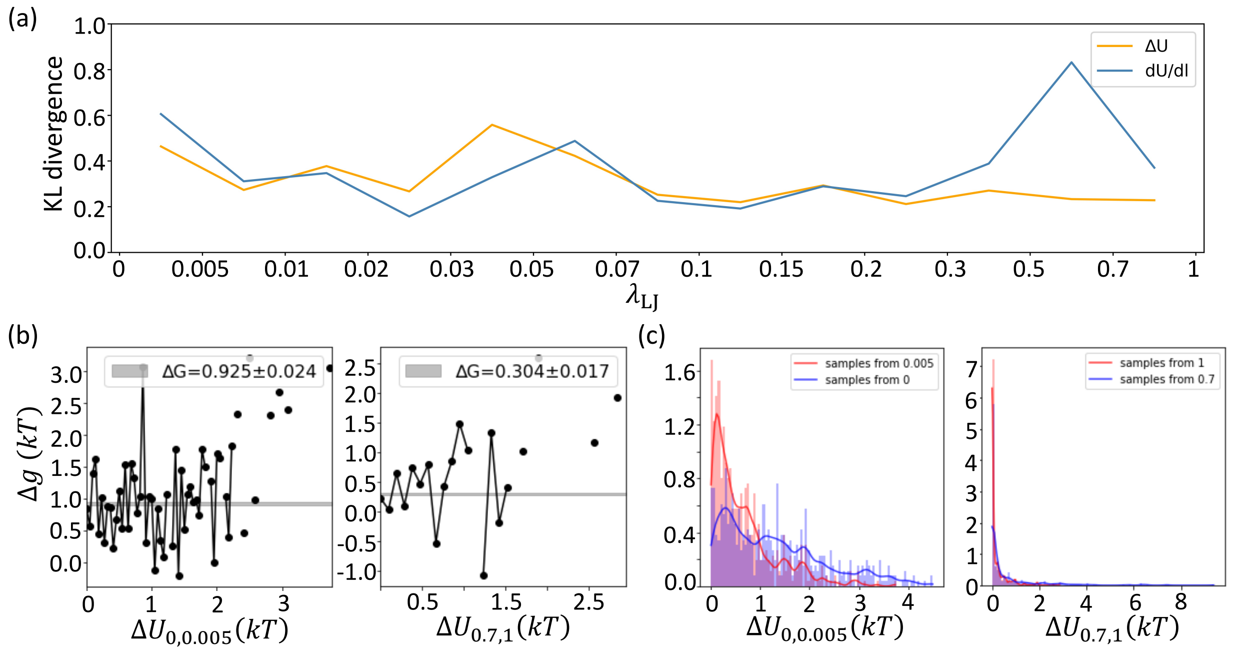

Figure S3: Phase space overlap in calculating $\Delta G^{\text{ternary(WCA)}}$ for BTK-CRBN in Fig. 2. **(a)** Overlap of ΔU and $\partial U/\partial\lambda$ distributions between adjacent states are quantified by the KL divergence. **(b)** Example Bennett's overlapping plots for $\lambda_{\text{LJ}} = 0, 0.005$ states (left) and $\lambda_{\text{LJ}} = 0.7, 1$ states (right). The grey bands represent $\Delta G_{\lambda_i,\lambda_{i+1}}$ ± 1 std estimated using BAR. **(c)** Example distributions of $\Delta U_{i,i+1}$ are shown with Gaussian smoothing (red and blue solid curves) for better visualization.

from a sufficient number of samples. The autocorrelation times all plummet to 0 before 0.63 s (Fig. S4b). Both equilibration time and decorrelation time are longer for simulations in lower value of λ_{LJ} states that retain more memory of previously sampled configurations due to lower energetic costs. Currently, the equilibration and autocorrelation cutoffs depend on each system. For convenience, we used the same cutoffs for all λ states. In the future, this can be customized for each state to maximize the number of samples, especially from states of high λ values that requires less equilibration and decorrelation time (Fig. S4b).

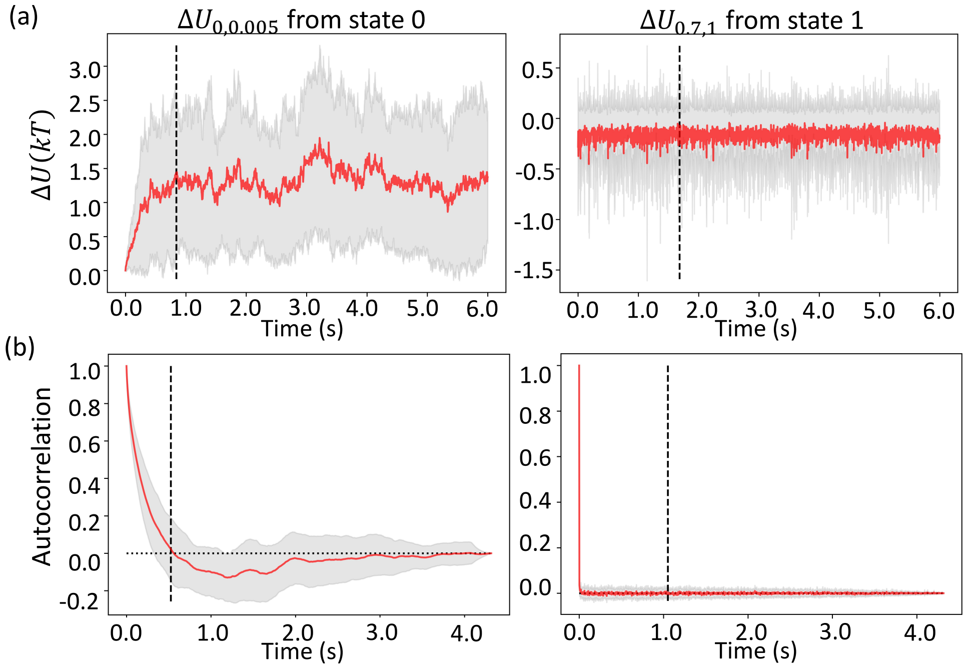

Figure S4: Detecting equilibration and autocorrelation time in calculating $\Delta G^{\mathrm{ternary(WCA)}}$ for BTK-CRBN in Fig. 2. **(a)** $\Delta U_{\lambda_i,\lambda_{i+1}}$ over simulation time and **(b)** the autocorrelation of $\Delta U_{\lambda_i,\lambda_{i+1}}$ from $\lambda_{\mathrm{LJ}} = 0$ (left) and $\lambda_{\mathrm{LJ}} = 1$ (right). The red curves and the shaded regions represent the average value ±1 standard deviation based on 64 independent trajectories. The vertical dashed lines in this example mark 0.9 s in (a) and 0.63 s in (b). The horizontal dotted lines in (b) mark the 0 autocorrelation value.

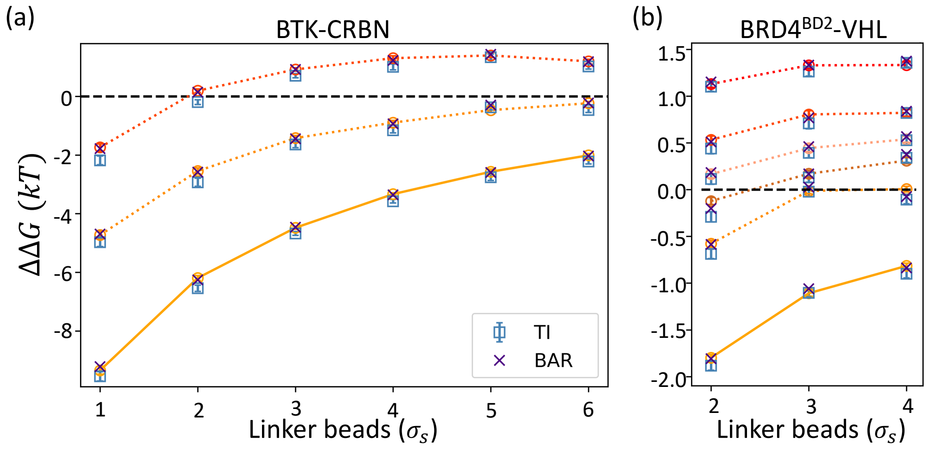

Figure S5: $\Delta\Delta G$s calculated by TI and BAR are superimposed onto the MBAR results shown in Figure 3 to show that all three alchemical free energy calculation methods agree within noise.

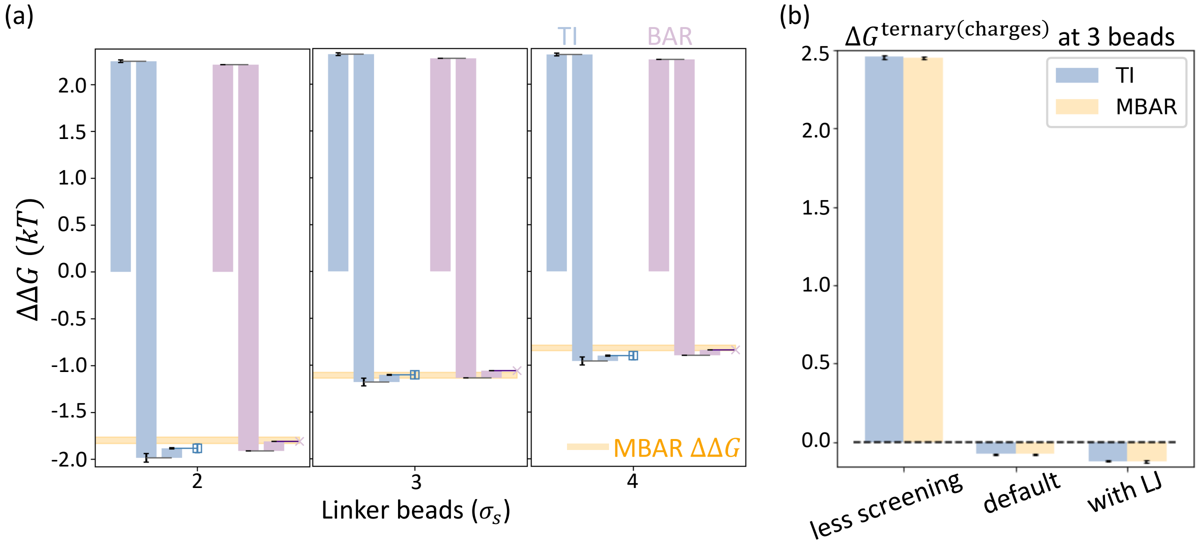

Figure S6: $\Delta\Delta G$s calculated by TI and BAR agree with MBAR results shown in Figure 4 for the BRD4$^{\text{BD2}}$-VHL system modeled with protein charges included. **(a)** $\Delta\Delta G$s at each PROTAC linker length calculated by TI and BAR are broken down using waterfall plots similar to Figure 4b. In each triplet, columns from left to right correspond to ΔG^{binary}, $-\Delta G^{\text{ternary(other)}}$, and $-\Delta G^{\text{ternary(charges)}}$. Columns are arranged cumulatively such that the end point of a triplet of columns represent the final $\Delta\Delta G$ value calculated by the corresponding method. MBAR $\Delta\Delta G$ values with ± 1 standard deviation are shown as horizontal yellow bands for reference. **(b)** TI and MBAR calculations of the electrostatic contribution to $\Delta\Delta G$ under different forcefield setups at the linker length of 3 beads agree with each other. Note that $\Delta G^{\text{ternary(charges)}}$ is shown here rather than $-\Delta G^{\text{ternary(charges)}}$ in panel **(a)**.

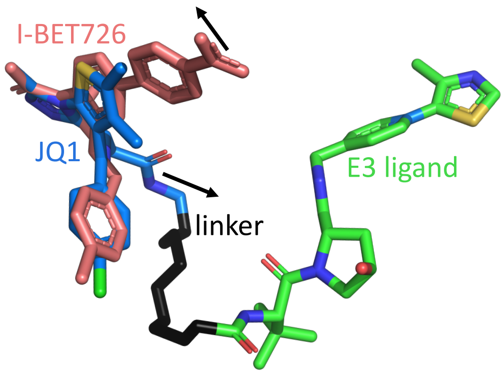

Figure S7: The structure of MZ1, which is a PROTAC with linker length of 3 beads using a JQ1 warhead, extracted from the ternary crystal structure (PDB: 5T35[1]) and the structure of I-BET726 warhead extracted from the crystal structure of a binary complex (PDB: 4BJX[14]) are superimposed to highlight the difference in exit vectors (black arrows).

References

(1) Gadd, M. S.; Testa, A.; Lucas, X.; Chan, K.-H.; Chen, W.; Lamont, D. J.; Zengerle, M.; Ciulli, A. Structural basis of PROTAC cooperative recognition for selective protein degradation. *Nature Chemical Biology* **2017**, *13*, 514–521, Number: 5 Publisher: Nature Publishing Group.

(2) Nowak, R. P.; DeAngelo, S. L.; Buckley, D.; He, Z.; Donovan, K. A.; An, J.; Safaee, N.; Jedrychowski, M. P.; Ponthier, C. M.; Ishoey, M.; Zhang, T.; Mancias, J. D.; Gray, N. S.; Bradner, J. E.; Fischer, E. S. Plasticity in binding confers selectivity in ligand-induced protein degradation. *Nature Chemical Biology* **2018**, *14*, 706–714, Number: 7 Publisher: Nature Publishing Group.

(3) Schiemer, J.; Horst, R.; Meng, Y.; Montgomery, J. I.; Xu, Y.; Feng, X.; Borzilleri, K.; Uccello, D. P.; Leverett, C.; Brown, S.; Che, Y.; Brown, M. F.; Hayward, M. M.; Gilbert, A. M.; Noe, M. C.; Calabrese, M. F. Snapshots and ensembles of BTK and cIAP1 protein degrader ternary complexes. *Nature Chemical Biology* **2021**, *17*, 152–160, Number: 2 Publisher: Nature Publishing Group.

(4) Madhavi Sastry, G.; Adzhigirey, M.; Day, T.; Annabhimoju, R.; Sherman, W. Protein and ligand preparation: parameters, protocols, and influence on virtual screening enrichments. *Journal of Computer-Aided Molecular Design* **2013**, *27*, 221–234.

(5) Niesen, M. J. M.; Wang, C. Y.; Lehn, R. C. V.; Miller, T. F. Structurally detailed coarse-grained model for Sec-facilitated co-translational protein translocation and membrane integration. *PLOS Computational Biology* **2017**, *13*, e1005427, Publisher: Public Library of Science.

(6) Liwo, A.; Ołdziej, S.; Pincus, M. R.; Wawak, R. J.; Rackovsky, S.; Scheraga, H. A. A united-residue force field for off-lattice protein-structure simulations. I. Functional forms and parameters of long-range side-chain interaction potentials from protein crystal data. *Journal of Computational Chemistry* **1997**, *18*, 849–873.

(7) Lezon, T. R.; Shrivastava, I. H.; Yang, Z.; Bahar, I. *Handbook on Biological Networks*; World Scientific Lecture Notes in Complex Systems Volume 10; WORLD SCIENTIFIC, 2009; Vol. Volume 10; pp 129–158.

(8) Ricardo Batista, P.; Herbert Robert, C.; Maréchal, J.-D.; Ben Hamida-Rebaï, M.; Geraldo Pascutti, P.; Mascarello Bisch, P.; Perahia, D. Consensus modes, a robust description of protein collective motions from multiple-minima normal mode analysis—application to the HIV-1 protease. *Physical Chemistry Chemical Physics* **2010**, *12*, 2850–2859, Publisher: Royal Society of Chemistry.

(9) Periole, X.; Cavalli, M.; Marrink, S.-J.; Ceruso, M. A. Combining an Elastic Network With a Coarse-Grained Molecular Force Field: Structure, Dynamics, and Intermolecular Recognition. *Journal of Chemical Theory and Computation* **2009**, *5*, 2531–2543, Publisher: American Chemical Society.

(10) Sievers, Q. L.; Petzold, G.; Bunker, R. D.; Renneville, A.; Słabicki, M.; Liddicoat, B. J.; Abdulrahman, W.; Mikkelsen, T.; Ebert, B. L.; Thomä, N. H. Defining the human C2H2

zinc finger degrome targeted by thalidomide analogs through CRBN. *Science* **2018**, *362*, eaat0572, Publisher: American Association for the Advancement of Science.

(11) Pohorille, A.; Jarzynski, C.; Chipot, C. Good Practices in Free-Energy Calculations. *The Journal of Physical Chemistry B* **2010**, *114*, 10235–10253, Publisher: American Chemical Society.

(12) Bennett, C. H. Efficient estimation of free energy differences from Monte Carlo data. *Journal of Computational Physics* **1976**, *22*, 245–268.

(13) Klimovich, P. V.; Shirts, M. R.; Mobley, D. L. Guidelines for the analysis of free energy calculations. *Journal of Computer-Aided Molecular Design* **2015**, *29*, 397–411.

(14) Wyce, A.; Ganji, G.; Smitheman, K. N.; Chung, C.-w.; Korenchuk, S.; Bai, Y.; Barbash, O.; Le, B.; Craggs, P. D.; McCabe, M. T.; Kennedy-Wilson, K. M.; Sanchez, L. V.; Gosmini, R. L.; Parr, N.; McHugh, C. F.; Dhanak, D.; Prinjha, R. K.; Auger, K. R.; Tummino, P. J. BET Inhibition Silences Expression of MYCN and BCL2 and Induces Cytotoxicity in Neuroblastoma Tumor Models. *PLOS ONE* **2013**, *8*, e72967, Publisher: Public Library of Science.
